# 600 Assessing the Need for Inclusion of Race or Ethnicity in the Modified Baux Score

**DOI:** 10.1093/jbcr/iraf019.229

**Published:** 2025-04-01

**Authors:** Dharani Rao, Cailan Feingold, Priscilla Tran, Kerri Finnan, Gabriel Rodriguez, Rachelle Lodescar

**Affiliations:** Burn Center at Westchester Medical Center; Burn Center at Westchester Medical Center; Burn Center at Westchester Medical Center; Burn Center at Westchester Medical Center; Westchester Medical Center; Burn Center at Westchester Medical Center

## Abstract

**Introduction:**

The revised Baux score (%TBSA + age + [17xR], R representing 1 or 0 based on the presence of inhalation injury), is a highly accurate predictor of mortality in burn patients, shown to be consistent across heterogeneous populations despite not accounting for individual patient characteristics. However, differences in outcomes on the basis of race, gender, and socioeconomic status among other variables suggests that the predictive value of models that do not account for these factors may have greater limitations than previously assessed. Further investigation reveals that variables not included in the Baux score calculation bear significant individual influence on prognosis and mortality for burn patients such as gender, race, mechanism of burn, socioeconomic status, and marginalized status. Therefore, we aim to assess the predictive accuracy of the revised Baux score among different racial/ethnic groups and, investigate whether racial differences among massive burn patients (>20% TBSA) are associated with in-hospital mortality differences after adjusting for confounding variables.

**Methods:**

We analyzed data from 92 patients, comparing those who died during hospitalization (N=31) with those who were discharged alive (N=61). The characteristics of the two groups were assessed using Wilcoxon rank-sum and chi-squared tests. A logistic regression model was used to adjust for potential confounders. Receiver operating characteristic (ROC) curves were constructed to evaluate Baux score performance for different racial/ethnic groups.

**Results:**

Patients who died were older (median age 59 years vs. 39 years, p< 0.001), had higher modified Baux scores (median 127 vs. 78, p< 0.001), greater total body surface area (TBSA) burns (median 60% vs. 29%, p< 0.001), and shorter hospital stays (median 3 days vs. 30 days, p< 0.001). Logistic regression identified a significant association between the modified Baux score (OR=1.06, 95% CI: 1.03-1.09, p< 0.001) and mortality, however racial/ethnic differences were not significant after adjustment (Black or Hispanic OR=0.41, 95% CI: 0.05-2.27, p=0.3; Asian OR=1.38, 95% CI: 0.22-7.99, p=0.7). ROC analysis showed that the overall area under the curve (AUC) for the model was 0.867: White patients had an AUC of 0.836, Black and Hispanic patients had an AUC of 0.922, and Asian patients had an AUC of 0.889.

**Conclusions:**

After adjusting for confounding factors, there was no significant difference in the Baux score or odds of mortality between racial/ethnic groups. The modified Baux score demonstrated good overall predictive accuracy for mortality regardless of race, with particularly high performance for Black and Hispanic patients, suggesting that inclusion of race into score calculation is not required.

**Applicability of Research to Practice:**

This work supports continued use of the Baux score as a predictive tool in the clinical setting when assessing mortality risk among increasingly diverse populations of adult burn patients.

**Funding for the Study:**

N/A

